# Clinical and prognostic significance of emergency MRI findings in neck infections

**DOI:** 10.1007/s00330-021-08200-5

**Published:** 2021-07-30

**Authors:** Jaakko Heikkinen, Janne Nurminen, Jarno Velhonoja, Heikki Irjala, Tatu Happonen, Tero Soukka, Kimmo Mattila, Jussi Hirvonen

**Affiliations:** 1 Department of Radiology, Turku University Hospital, Kiinamyllynkatu 4-8, 20521 Turku, Finland; 2 Department of Otorhinolaryngology-Head and Neck Surgery, University of Turku and Turku University Hospital, Kiinamyllynkatu 4-8, FI-20520 Turku, Finland; 3 Department of Oral and Maxillofacial Surgery, University of Turku, Turku, Finland; 4 Department of Radiology, University of Turku, Turku, Finland

**Keywords:** Magnetic resonance imaging, Neck, Emergency medicine, Infection

## Abstract

**Objectives:**

Due to its superior soft-tissue contrast and ability to delineate abscesses, MRI has high diagnostic accuracy in neck infections. Whether MRI findings can predict the clinical course in these patients is unknown. The purpose of this study was to determine the clinical and prognostic significance of various MRI findings in emergency patients with acute neck infections.

**Materials and methods:**

We retrospectively reviewed the 3-T MRI findings of 371 patients with acute neck infections from a 5-year period in a single tertiary emergency radiology department. We correlated various MRI findings, including retropharyngeal (RPE) and mediastinal edema (ME) and abscess diameter, to clinical findings and outcomes, such as the need for intensive care unit (ICU) treatment and length of hospital stay (LOS).

**Results:**

A total of 201 out of 371 patients (54%) with neck infections showed evidence of RPE, and 81 out of 314 patients (26%) had ME. Both RPE (OR = 9.5, *p* < 0.001) and ME (OR = 5.3, *p* < 0.001) were more prevalent among the patients who required ICU treatment than among those who did not. In a multivariate analysis, C-reactive protein (CRP) levels, RPE, and maximal abscess diameter were independent predictors of the need for ICU treatment, and CRP, ME, and maximal abscess diameter were independent predictors of LOS.

**Conclusion:**

In patients with an acute neck infection that requires emergency imaging, RPE, ME, and abscess diameter, as shown by MRI, are significant predictors of a more severe illness.

**Key Points:**

*• Two hundred one out of 371 patients (54%) with neck infection showed evidence of retropharyngeal edema (RPE), and 81 out of 314 patients (26%) had mediastinal edema (ME).*

*• Maximal abscess diameter, RPE, and C-reactive protein (CRP) were independent predictors of the need for intensive care unit (ICU) treatment, and maximal abscess diameter, ME, and CRP were independent predictors of length of hospital stay.*

*• Prognostic significance of MRI findings was evident also while controlling for CRP values.*

## Introduction

Deep neck infections present challenges, even in modern medicine, due to their complex anatomy and potentially lethal complications [1]. The true extent of the infection is difficult to assess clinically. Therefore, emergency imaging is often required to determine the exact location and extent of the disease. In suspected neck infection cases, CT has traditionally been the first-line imaging method [2, 3]. However, distinguishing between reactive non-suppurative edema and true abscesses in deep neck spaces using CT may be challenging [4–6]. This distinction is critical for choosing the appropriate treatment: abscesses usually require surgical drainage, whereas infections without abscesses usually resolve after conservative treatment. MRI provides excellent soft-tissue characterization, surpassing that of CT in the imaging evaluation of neck infections [7]. Although MRI is often considered time consuming and challenging for acutely ill patients, emergency neck MRI has recently shown to be feasible and to have superior diagnostic accuracy to that previously reported for CT [8].

The retropharyngeal space is a deep neck space between the visceral fascia anteriorly and prevertebral fascia posteriorly. This space is further divided by the thin alar fascia to the true retropharyngeal space anteriorly and the danger space posteriorly. The true retropharyngeal space extends from the skull base to the upper mediastinum, whereas the danger space extends further down to the level of the diaphragm [9]. Thus, both the true retropharyngeal space and the danger space may serve as conduits of disease progression from the neck to the mediastinum, although they cannot be reliably separated using imaging. Reactive, non-suppurative retropharyngeal edema and fluid collections have been previously described anecdotally and in case studies in the literature in various neck diseases, including infection [10–13]. In addition, in clinical practice, subtle edema is often seen to reach the mediastinum in various neck infections, already in the early stages of the disease. Currently, the clinical significance of these MRI edema patterns is unknown.

This study aimed to investigate the nature and prevalence of MRI edema patterns in patients with neck infections. We focused on maximal abscess diameter and two specific edema patterns: retropharyngeal edema (RPE) and mediastinal edema (ME). The clinical and prognostic values of these findings were assessed in terms of outcomes related to the severity of the disease, including intensive care unit (ICU) admissions and length of hospital stay (LOS).

## Material and methods

### Patients

We retrospectively reviewed patients who had undergone emergency MRI for suspected neck infection between April 1, 2013, and December 31, 2018, in a single academic tertiary care referral center. We obtained permission from the hospital district board, and written patient consent was not obtained due to the retrospective nature of the study. Institutional review board (IRB) review (approval or waiver) was not sought, because it is not required by the national legislature for retrospective studies of existing data. Neck MRI was performed on these patients due to clinical suspicion of severe or deep neck infection. The inclusion criteria were (1) emergency MRI evidence of infection: high signal of fat-suppressed T2-weighted Dixon images suggesting edema, or high signal of fat-suppressed post-Gadolinium (post-Gd) T1-weighted Dixon images suggesting abnormal tissue enhancement; (2) final clinical diagnosis of infection; and (3) diagnostic image quality as deemed by the radiologist reading the study. Origin of the infection was determined as the final clinical diagnosis from the medical records. MRI was performed prior to hospital admission at the emergency radiology department using a Philips Ingenia 3-T system with a dS HeadNeckSpine coil configuration (Philips Healthcare). Details of the MRI are provided in the Supplementary information. We routinely administered a Gd-based contrast agent (gadoterate meglumine, Dotarem®, Guerbet). Demographic, clinical, and laboratory variables were extracted from medical records: age (years), sex (male/female), body mass index (BMI, kg/m^2^), duration of symptoms prior to imaging (days), C-reactive protein (CRP, mg/L), white blood cell count (WBC, × 10^9^/L), body temperature (°C), surgery (yes/no), treatment in an ICU (yes/no), and LOS (days). The need for ICU treatment was determined only on clinical grounds, e.g., to secure adequate ventilation or vital functions. Laboratory values were taken on admission or, in the case of multiple assessments, the most recent prior to imaging.

### Definition of imaging outcomes and ratings

The main location of the infection was tabulated, as per final clinical diagnosis. In addition, we pooled two major groups of infections separately: pharyngotonsillar (including tonsillitis, peritonsillar abscess, and parapharyngeal space involvement) and odontogenic infections (mostly oral cavity, masticator, and buccal spaces, with a clinical confirmation of odontogenic origin by an oral surgeon).

We defined an abscess as an abnormal T2-hyperintense collection with a low apparent diffusion coefficient (ADC) surrounded by abnormal tissue enhancement on post-Gd T1-weighted images and no enhancement in the center. In analyses including abscess measurements, we excluded patients in whom purulence or an abscess cavity could not be surgically demonstrated (false positives, *N* = 14). We measured the maximal diameter of the abscesses from post-Gd T1-weighted images. Substantial interobserver agreement for the detection of an abscess has been shown previously [8].

Retropharyngeal edema (RPE) was defined as a hyperintense signal of at least 2 mm in anteroposterior thickness between the prevertebral muscles posteriorly and the superior pharyngeal constrictor muscle anteriorly, in at least two consecutive axial fat-suppressed T2-weighted Dixon images. Within the RPE, we further defined enhancing phlegmon as hyperintense areas on post-Gd T1-weighted Dixon images, and free fluid as any non-enhancing hypointense areas on post-Gd T1-weighted Dixon images within the phlegmon tissue. Free fluid was deemed purulent if it had a low apparent diffusion coefficient (ADC) and non-purulent if it had a high ADC on visual inspection. At the level of maximal RPE, we measured the thickness (mm) and craniocaudal position relative to the cervical vertebrae. We also recorded whether the RPE was suprahyoid, infrahyoid, or both.

Mediastinal edema (ME) was defined as a hyperintense signal in the soft tissues in axial or coronal fat-suppressed T2-weighted Dixon images at or below the level of the thoracic inlet, using the superior border of the manubrium sterni (anterior ME), or the first thoracic vertebra (posterior ME) as superior borders. Anterior ME was often contiguous with edema in the visceral space, while posterior ME was often a continuation of infrahyoid RPE.

RPE, ME, and abscess diameter were defined by a board-certified radiologist, fellowship trained in both neuroradiology and head and neck radiology (J.Hi., 10 years of experience in diagnostic radiology). To ensure the reliability of these assessments, another board-certified radiologist (J.N., 8 years of experience in diagnostic radiology) independently evaluated images for three different findings (RPE, ME, abscess diameter). For each finding, images from a random sample of 60 patients were evaluated, and each of these random samples was evaluated on separate occasions.

### Statistical analyses

The results are expressed as percentages, means, and standard deviation (SD). For univariate assessments, we used Pearson *T* tests for continuous data and the chi square (*Χ*^2^) test and odds ratios (OR) for ordinal data. We evaluated inter-rater reliability using Cohens’ kappa for ordinal data and an intraclass correlation coefficient (ICC) for continuous data. In univariate analyses, we included age, sex, BMI, duration of symptoms, CRP, and WBC as clinical and laboratory parameters of interest. Body temperature was not included because of a large amount of missing data. For multivariate prediction models, we used binary logistic regression for dichotomous outcomes and linear regression for continuous outcomes. In these models, we only included variables that had been significant at the univariate level. We constructed three different models for both ICU treatment and LOS, in order to compare the added value of MRI findings. Model 1 had only significant clinical or laboratory predictors (no MRI findings). Model 2 added MRI edema patterns, and model 3 added abscesses. To make the models comparable, all had similar sample sizes. Model 1 only included patients in whom MRI edema patterns were available for analysis. Furthermore, in model 3, patients with no MRI evidence of abscess or false-positive abscesses in surgery were assigned “0” for maximal abscess diameter. In the regression models, all the predictors were entered into the model (fixed predictors). *R*^2^ values were compared between models. Because of the retrospective nature of the study, the sample size varies in different statistical analyses depending on which data is available. *p* values less than 0.05 were considered statistically significant. We analyzed the data using IBM SPSS Statistics for Mac (version 26, copyright IBM Corporation 2019).

## Results

### Patients and types of infections

**Fig. 1:**
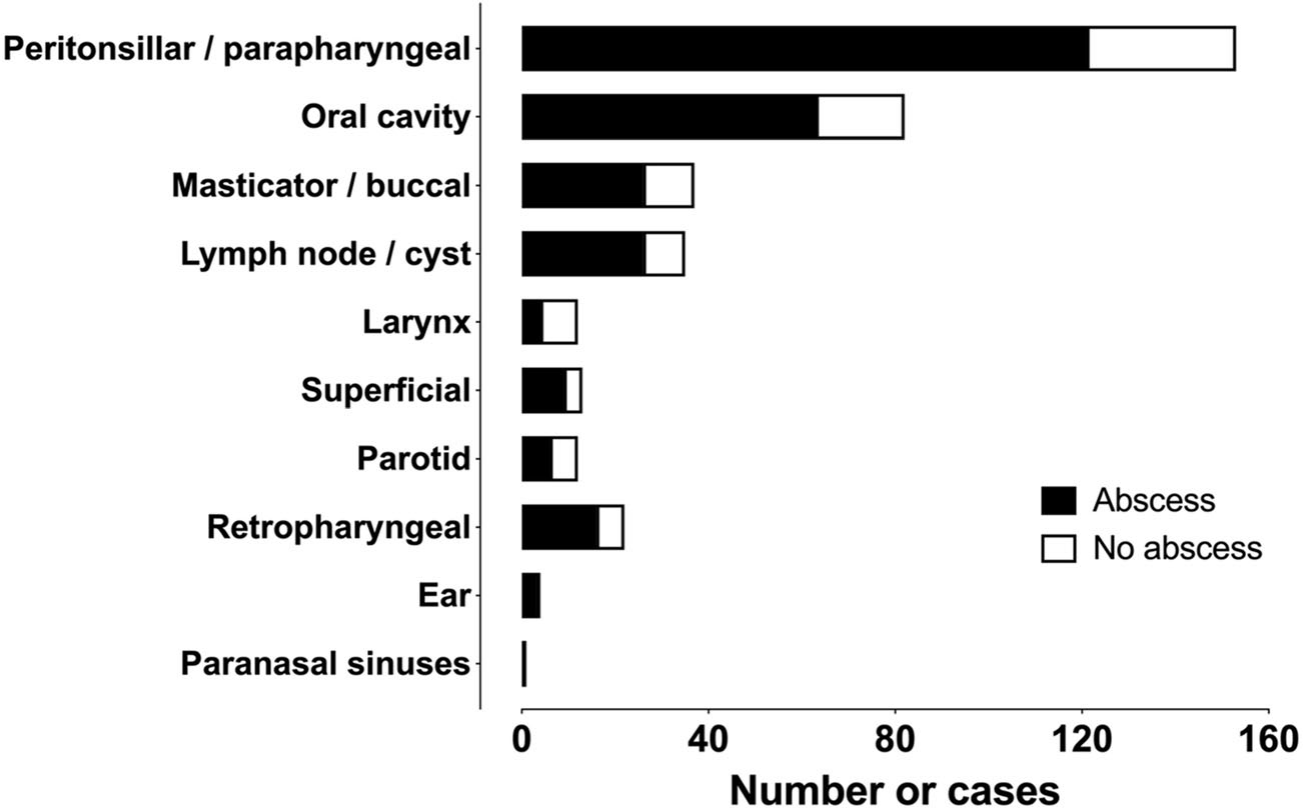
Anatomical localization of neck infections

Our study included 371 patients (226 male, 145 female) with clinically confirmed acute neck infection (Table 1). The patients had a mean age of 41 (range 0–88 years). Pediatric patients (<18 years old) represented 12% (*N* = 45) of the total sample. The mean duration of symptoms before imaging was about 5 days. As many as 254 (69%) patients underwent surgery, and 50 (14%) patients were treated at the ICU. Mean LOS was 4 days (range 0–55). The two most common types of infection were pharyngotonsillar (36%) and odontogenic (29%), followed by other less common locations (35%) (Fig. 1).

**Table 1:** Patient characteristics

Characteristic	
Number of patients	371
Age (years, mean ± SD)	41 ± 20
Male (*N*, %)	226 (61%)
Female (*N*, %)	145 (39%)
BMI (kg/m^2^)	27.7
CRP (mg/L)	121^a^ ± 86
WBC (× 10^9^/L)	14.6^b^ ± 12
Body temperature (°C)	37.5^c^ ± 0.8
Duration of symptoms before imaging (days)	5.0^d^ ± 4.4
ICU	50 (14%)
Length of hospital stay (LOS) (days)	4.4 ± 5.4^e^
Surgery	254 (69%)

Data available for (a) 364, (b) 362, (c) 250, (d) 363, and (e) 364 patients; LOS could not be determined in 7 patients because of transfers to other hospitals. Seventeen patients (5%) were not hospitalized

### Imaging patterns

**Fig. 2:**
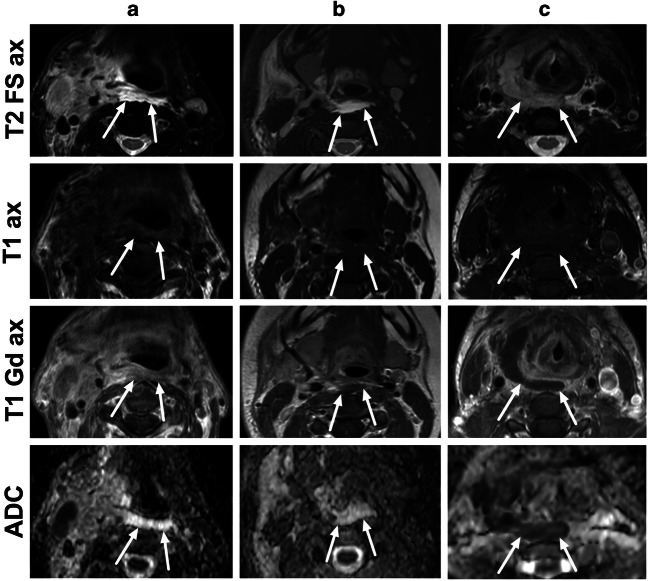
Different types of RPE in axial fat-suppressed T2-weighted Dixon images (first row), axial pre-contrast T1-weighted images (second row), axial post-contrast in-phase Dixon T1-weighted images (third row), and axial ADC maps (fourth row), from three patients (**a**–**c**). **a** A 63-year-old male with tonsillitis had high T2 signal, contrast enhancement on T1, and no purulence on ADC (enhancement-type RPE). **b** A 23-year-old female with parotitis had high T2-signal, non-enhancing fluid on T1, and no purulence on ADC (fluid-type RPE). **c** A 41-year-old female with a throat infection had an intermediate T2-signal, non-enhancing fluid on T1, and low ADC consistent with purulent fluid (true retropharyngeal abscess)

**Fig. 3:**
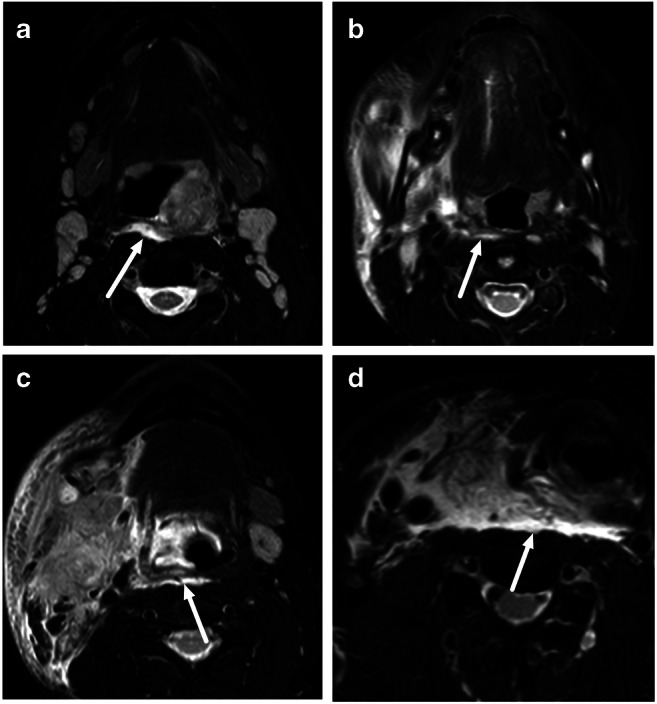
Examples of RPE on axial fat-suppressed T2-weighted Dixon images. **a** A 17-year-old male with left-sided peritonsillar infection and abscess. **b** A 13-year-old male with right-sided odontogenic infection. **c** An 18-year-old male with superficial (lateral) lymphadenitis. **d** A 59-year-old male with infected thyroid mass (papillary carcinoma)

RPE was found in 201 (54%) patients (Table 2). One hundred forty-nine patients (74%) had retropharyngeal enhancement and 52 (26%) patients had free fluid collections (Fig. 2.). The mean thickness of RPE was 4.7 ± 2.3 (SD) mm. The thickest portion of RPE was most commonly at the C3 level (50%). Relative to the hyoid bone, 115 (57%) patients had RPE on both sides, superior and inferior to the hyoid bone, whereas 84 (42%) patients had suprahyoid only and 2 (1.0%) patients infrahyoid only. RPE was seen in various types of neck infections (Fig. 3).

**Table 2:** Imaging outcomes. Values are *N* (%) or mean ± SD

Outcome	
Abscess	260 (73%)^a^
Maximal abscess diameter (mm)	35 ± 22
RPE, presence	201 (54%)
Level (median)	C3 (50%)
Enhancing type	149 (74%)
Fluid type	52 (26%)
Thickness (mm)	4.7 ± 2.3
Suprahyoid	84 (42%)
Infrahyoid	2 (1.0%)
Both	115 (57%)
ME, presence	81 (26%)^b^
Anterior	48 (59%)
Posterior	39 (48%)
Both	6 (7.4%)

^a^True positives

^b^In whom ME could be evaluated (311 of 371 studies)

When comparing the two major groups of neck infection, we found that RPE was more prevalent among patients with pharyngotonsillar infection (76%), than among those with odontogenic infections (35%) (*p* < 0.001). RPE was also thicker in pharyngotonsillar infections (4.4 ± 1.9 mm) than in odontogenic infections (3.6 ± 1.1 mm). Pharyngotonsillar infections had greater caudal extension of RPE than odontogenic infections: 56% vs. 11% occurring at the C4–5 level and 60% vs. 22% extending below the hyoid bone, respectively.

**Fig. 4:**
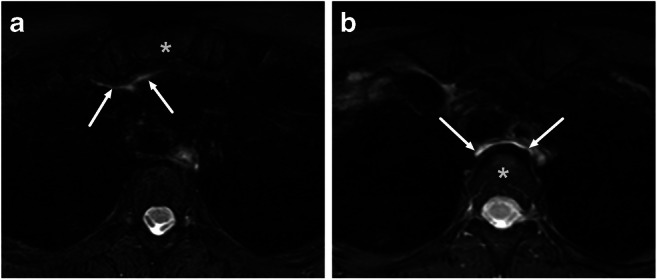
Examples of anterior (**a**) and posterior (**b**) mediastinal edema on axial fat-suppressed T2-weighted Dixon images. **a** A 46-year-old female with tonsillitis had edema in the anterior mediastinum at the level of the manubrium sterni (asterisk). **b** A 48-year-old female with peritonsillar and parapharyngeal abscesses had edema in the posterior mediastinum at the level of the Th3 vertebra (asterisk)

The images covered the upper mediastinum sufficiently to evaluate ME in 311 (84%) patients, among 81 of whom ME was present (26%): anterior in 59%, posterior in 48%, and both in 7.4% (Table 2, Fig. 4). ME was equally prevalent among pharyngotonsillar (15%) and odontogenic infections (23%), although it occurred mostly posteriorly in the former (88%) and anteriorly (95%) in the latter.

The two edema patterns, RPE and ME, tended to co-occur (*p* < 0.001); for example, ME occurred in 37% of patients with RPE and in 13% of those without. Adult and pediatric patients had similar rates of RPE and ME (Supplementary information).

Two hundred seventy-four patients had MRI evidence of an abscess, of whom 229 underwent surgery. Excluding 14 false-positive abscesses, data from 260 abscesses were analyzed. The mean maximal abscess diameter was 35 mm. No differences were found between the prevalence (85% vs. 76%) or maximal diameters (33 vs. 35 mm) of the abscesses in pharyngotonsillar and odontogenic infections (respectively).

RPE was significantly more prevalent in the patients with an abscess (62%) than in those without (34%) (*p* < 0.001), whereas ME was equally prevalent (27% vs. 22%, *p* = 0.332).

In the independent inter-rater evaluation of 60 randomly selected patients, imaging outcomes were reliable, as indicated by kappa values consistent with substantial agreement (0.781 and 0.675 for RPE and ME, respectively), and ICC values consistent with good reliability (0.81 and 0.75 for RPE thickness and maximal abscess diameter, respectively).

### Clinical and prognostic value of MRI findings

**Fig. 5:**
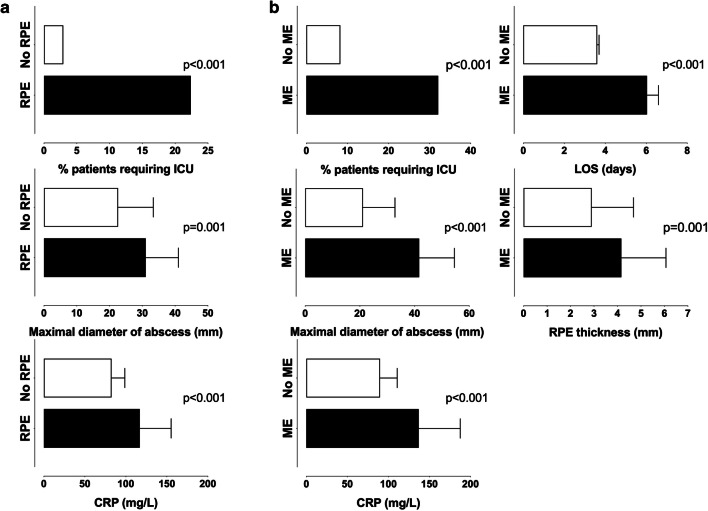
Clinical significance of RPE (**a**) and ME (**b**). Presence of RPE and ME were predictors of ICU treatment, larger abscesses, and higher CRP; ME was also a predictor of longer LOS and thicker RPE

Overall, the patients with RPE and ME had higher incidence of markers consistent with more severe disease, including higher incidence of ICU treatment, larger abscesses, higher CRP, and longer LOS, than those without (Fig. 5).

#### Prediction of ICU treatment

Retropharyngeal edema (RPE) was present in 90% of the patients (45/50) who required ICU treatment and in 49% of the patients (156/321) who did not (OR = 9.5, *p* < 0.001). Mediastinal edema (ME) was present in 58% of the patients (26/45) who required ICU treatment and in 21% of the patients (55/266) who did not (OR = 5.3, *p* < 0.001). These effects were similar in adult and pediatric patients (Supplementary information). RPE and ME were significantly associated with ICU treatment for odontogenic infections (*p* < 0.001 for both) but not in pharyngotonsillar infections (*p* = 0.060 and *p* = 0.096 for RPE and ME, respectively). Patients who required ICU treatment had higher CRP (188 vs. 111, *p* < 0.001), larger maximal abscess diameter (5.4 vs. 3.1 cm, *p* <0.001), and thicker RPE (5.4 vs. 4.5 mm, *p* = 0.050), whereas other clinical and laboratory variables were not significantly different. Comparing different prediction models revealed that inclusion of MRI findings improved the prediction capability of the multivariate models (Table 3). In 307 patients with MRI edema patterns and clinical data available for analysis, the need for ICU treatment was predicted by CRP (*p* = 0.015), RPE (*p* = 0.004), and maximal abscess diameter (*p* = 0.006) (Table 3, model 3).

**Table 3:** Multivariate models for predicting ICU treatment. Odds ratios, *p* values, and Nagelkerke *R*^2^ values from binary logistic regression analyses

	**Model 1**		**Model 2**		**Model 3**	
Variable	Odds ratio	*p* value	Odds ratio	*p* value	Odds ratio	*p* value
CRP	1.01	< 0.001	1.01	0.001	1.01	0.015
RPE			5.78	0.002	5.07	0.004
ME			2.64	0.011	2.09	0.067
Abscess diameter					1.02	0.006
Model *R*^2^	0.17		0.29		0.33	

Model 1 only has a clinical predictor, model 2 has a clinical predictor and MRI edema patterns, and model 3 adds the maximal abscess diameter in patients with an abscess. All models have 307 patients in whom ME could be evaluated and other data was available. In model 3, patients with no MRI evidence of abscess or false-positive abscesses in surgery were assigned “0” for maximal abscess diameter

#### Prediction of LOS

LOS was longer among those with ME than among those without (6.4 vs. 3.5 days, *p* < 0.001), but not significantly different between those with and without RPE (4.8 vs. 4.0 days, *p* = 0.149). The effect of ME on LOS was more pronounced in odontogenic (6.8 vs. 3.2 days, *p* = 0.025) than in pharyngotonsillar infections (5.9 vs. 3.0 days, *p* = 0.093). LOS was positively correlated with CRP (*p* < 0.001), WBC (*p* < 0.001), and maximal abscess diameter (*p* < 0.001), whereas other clinical and laboratory variables were not significantly associated with LOS. Inclusion of MRI findings improved the multivariate prediction models (Table 4). In 307 patients with MRI edema patterns and clinical data available, LOS was predicted by CRP (*p* < 0.001), ME (*p* = 0.012), and maximal abscess diameter (*p* < 0.001) (Table 4, model 3). These effects were similar in adult and pediatric patients (Supplementary information).

**Table 4:** Multivariate models for predicting LOS. Standardized beta values, *p* values, and *R*^2^ values from linear regression analyses

	**Model 1**		**Model 2**		**Model 3**	
Variable	Beta	*p* value	Beta	*p* value	Beta	*p* value
CRP	0.32	< 0.001	0.26	< 0.001	0.21	< 0.001
WBC	0.10	0.089	0.09	0.124	0.05	0.331
RPE			0.002	0.969	−0.03	0.657
ME			0.18	0.002	0.15	0.012
Abscess diameter					0.22	< 0.001
Model *R*^2^	0.13		0.16		0.20	

Model 1 only has clinical predictors, model 2 has clinical predictors and MRI edema patterns, and model 3 adds the maximal abscess diameter in patients with an abscess. All models have 307 patients in whom ME could be evaluated and other data was available. In model 3, patients with no MRI evidence of abscess or false-positive abscesses in surgery were assigned “0” for maximal abscess diameter

## Discussion

The primary importance of emergency imaging is to provide a correct diagnosis and an anatomical basis for the management of deep neck infections. In our study of 371 patients with emergency neck MRI, we found that patients with retropharyngeal edema (RPE) and mediastinal edema (ME) had a more severe course of illness, indicated by higher incidence of ICU treatment, larger abscesses, higher CRP, and longer LOS than those without these edema patterns. Conversely, the patients who required ICU treatment had a higher prevalence of RPE and ME than those who did not. RPE and ME were more strongly associated with ICU treatment among odontogenic infection cases than among pharyngotonsillar disease cases. LOS was 3 days longer among patients with ME than among those without, and this effect was most pronounced in cases of odontogenic infections. Notably, these MRI edema patterns were associated with ICU treatment and longer LOS while controlling for CRP. In patients with neck infections, MRI edema patterns can thus be used to predict more severe courses of illness.

The extent of non-suppurative reactive edema provided valuable information for predicting disease severity. Some of these edema patterns, such as RPE, have previously been described as case studies. We show that these patterns can be standardized to specific locations and measured with substantial reader agreement. Importantly, these edema patterns were found in various neck infections and not only in primarily retropharyngeal infections, which were rare (6.9%). RPE and ME should be considered imaging biomarkers of widespread edema rather than treatable retropharyngeal or mediastinal disease. They reflect the intensity of the disease, such that when the disease is more severe, it crosses the boundaries between soft-tissue compartments. These findings differed between types of infections: They were more significant in odontogenic than in pharyngotonsillar infections, reflecting the distance and barriers that the edema must exceed. Odontogenic infections often had anterior ME as continuous with anterior visceral space edema, while pharyngotonsillar infections were typically associated with posterior ME continuous with infrahyoid RPE. Whether edema spreads directly or via lymphatic spread is unclear.

Among patients with deep neck infections, CT with iodinated contrast has traditionally been the method of choice to evaluate the extent of the disease. Accurate evaluation becomes crucial for detecting abscesses, which usually require prompt surgical exploration. MRI was recently shown to be accurate in detecting abscesses that far exceed those previously reported for CT [8]. In the same study, the positive predictive value (PPV, fraction of patients with a positive test result who actually have the disease) for the detection of an abscess was 0.95, compared with about 0.80 previously reported for CT. Differentiation between RPE and true retropharyngeal abscess may be especially difficult with CT, with studies showing PPV in the range of 0.50 to 0.80 [4, 13–15]. Among children, no correlation was found between prevertebral soft-tissue thickening in CT and surgical incision and positive drainage [16]. This problem has also been described among patients with Kawasaki disease, for whom RPE is common and can be misdiagnosed as a retropharyngeal abscess [17]. We are unsure whether CT can measure RPE or ME to a similar extent and provide a prognostic value similar to MRI. A previous study using repeat CT showed that RPE improved after conservative treatment, despite the progression of the abscess itself, and did not indicate purulent extension outside the original abscess cavity [13]. The time course of the edema patterns in MRI is not yet known.

The strengths of the current study include its large sample size, systematic evaluation of the MRI findings, thorough clinical characterization, and surgical confirmation of abscesses. However, several limitations need to be addressed. Potential causes of bias include the fact that medical and surgical records may have been incomplete or imprecise, and that the caudal coverage of images was not always sufficient to assess ME. Our real-life cohort included both pediatric and adult patients, and similar results were obtained. We included patients with variable duration of symptoms before MRI, but the need for imaging was based on clinical grounds at time of presentation. Second, there are no universally accepted criteria for edema patterns, and interpretation may be subjective. However, we found substantial interobserver reliability in the MRI imaging patterns. Third, although MRI clearly shows edema, referring doctors or on-call radiologists may not be comfortable reading emergency neck MRI. Finally, regarding the generalizability of the current results, MRI may not always be suitable or available for all patients, and these results may not apply to all institutions. Patient management, including criteria for ICU admissions or surgery, may vary between hospitals. MRI costs more than CT, but this may be mitigated by improved PPV and potential prevention of unnecessary surgery.

In conclusion, reactive non-suppurative edema and abscess diameter on neck MRI were independent predictors of a severe course of illness among patients with neck infections. These findings suggest that emergency neck MRI could have clinical added value for compatible patients.

## Abbreviations

ICC: Intraclass correlation coefficient
ICU: Intensive care unit
LOS: Length of hospital stay
ME: Mediastinal edema
OR: Odds ratio
RPE: Retropharyngeal edema

## Declarations

### Guarantor

The scientific guarantor of this publication is Dr. Jussi Hirvonen.

### Statistics and biometry

No complex statistical methods were necessary for this paper.

### Methodology

*•* retrospective

*•* cross-sectional study

*•* performed at one institution
